# Efficacy and safety of pulmonary vein isolation with pulsed field ablation vs. novel cryoballoon ablation system for atrial fibrillation

**DOI:** 10.1093/europace/euad329

**Published:** 2023-11-30

**Authors:** Patrick Badertscher, Simon Weidlich, Sven Knecht, Niklas Stauffer, Philipp Krisai, Gian Voellmin, Stefan Osswald, Christian Sticherling, Michael Kühne

**Affiliations:** Department of Cardiology, University Hospital Basel, Petersgraben 4, 4031 Basel, Switzerland; Cardiovascular Research Institute Basel, University Hospital Basel, Spitalstrasse 2, 4056 Basel, Switzerland; Department of Cardiology, University Hospital Basel, Petersgraben 4, 4031 Basel, Switzerland; Cardiovascular Research Institute Basel, University Hospital Basel, Spitalstrasse 2, 4056 Basel, Switzerland; Department of Cardiology, University Hospital Basel, Petersgraben 4, 4031 Basel, Switzerland; Cardiovascular Research Institute Basel, University Hospital Basel, Spitalstrasse 2, 4056 Basel, Switzerland; Department of Cardiology, University Hospital Basel, Petersgraben 4, 4031 Basel, Switzerland; Cardiovascular Research Institute Basel, University Hospital Basel, Spitalstrasse 2, 4056 Basel, Switzerland; Department of Cardiology, University Hospital Basel, Petersgraben 4, 4031 Basel, Switzerland; Cardiovascular Research Institute Basel, University Hospital Basel, Spitalstrasse 2, 4056 Basel, Switzerland; Department of Cardiology, University Hospital Basel, Petersgraben 4, 4031 Basel, Switzerland; Cardiovascular Research Institute Basel, University Hospital Basel, Spitalstrasse 2, 4056 Basel, Switzerland; Department of Cardiology, University Hospital Basel, Petersgraben 4, 4031 Basel, Switzerland; Cardiovascular Research Institute Basel, University Hospital Basel, Spitalstrasse 2, 4056 Basel, Switzerland; Department of Cardiology, University Hospital Basel, Petersgraben 4, 4031 Basel, Switzerland; Cardiovascular Research Institute Basel, University Hospital Basel, Spitalstrasse 2, 4056 Basel, Switzerland; Department of Cardiology, University Hospital Basel, Petersgraben 4, 4031 Basel, Switzerland; Cardiovascular Research Institute Basel, University Hospital Basel, Spitalstrasse 2, 4056 Basel, Switzerland

**Keywords:** Atrial fibrillation, Pulsed field ablation, Cryoballoon, Pulmonary vein isolation

## Abstract

**Aims:**

Pulsed-field ablation (PFA) has emerged as a novel treatment technology for patients with atrial fibrillation (AF). Cryoballoon (CB) is the most frequently used single shot technology. A direct comparison to a novel CB system is lacking. We aimed to compare pulmonary vein isolation (PVI) using PFA vs. a novel CB system regarding efficiency, safety, myocardial injury, and outcomes.

**Methods and results:**

One hundred and eighty-one consecutive patients underwent PVI and were included (age 64 ± 9.7 years, ejection fraction 0.58 ± 0.09, left atrial size 40 ± 6.4 mm, paroxysmal AF 64%). 106 patients (59%) underwent PFA (FARAPULSE, Boston Scientific) and 75 patients (41%) underwent CB ablation (PolarX, Boston Scientific). The median procedure time, left atrial dwell time and fluoroscopic time were similar between the PFA and the CB group with 55 [interquartile range (IQR) 43–64] min vs. 58 (IQR 48–69) min (*P* < 0.087), 38 (30–49) min vs. 37 (31–48) min, (*P* = 0.871), and 11 (IQR 9.3–14) min vs. 11 (IQR 8.7–16) min, (*P* < 0.81), respectively. Three procedural complications were observed in the PFA group (two tamponades, one temporary ST elevation) and three complications in the CB group (3× reversible phrenic nerve palsies). During the median follow-up of 404 days (IQR 208–560), AF recurrence was similar in the PFA group and the CB group with 24 vs. 30%, *P* = 0.406.

**Conclusion:**

Procedural characteristics were very similar between PFA and CB in regard to procedure duration fluoroscopy time and complications. Atrial fibrillation free survival did not differ between the PFA and CB groups.

## Graphical Abstract

**Graphical Abstract:**
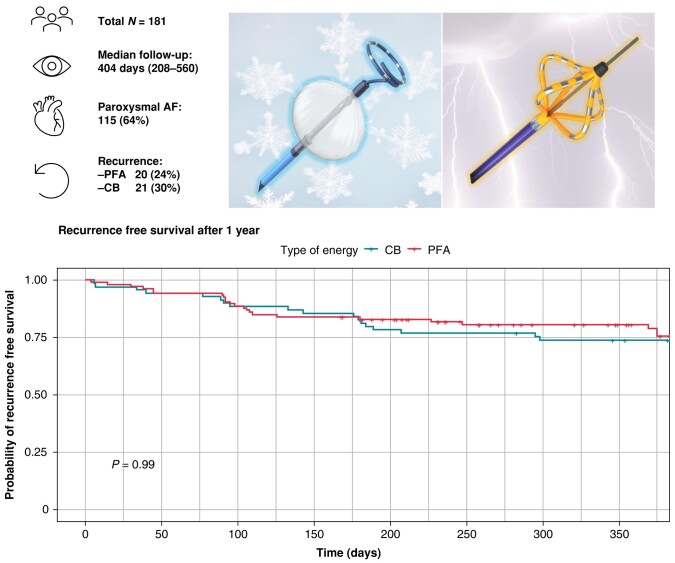
Comparison between pulsed field ablation and cryoballoon PolarX. AF, atrial fibrillation.

**What’s new?**

- This study is the first comparison of a novel cryoballoon (CB) ablation system and pulsed field ablation (PFA) in a large cohort of patients.
- Atrial fibrillation free survival after 1 year was ∼75% and rates were comparable for both technologies.
- Safety was similar in both groups, but phrenic nerve palsy only occurred in the CB group.
- Procedural efficiency was high for PFA and CB ablation and decreased over time with a median procedure time of <1 h in both groups.

## Introduction

Atrial fibrillation (AF) is the most common arrhythmia and contributes significantly to patient morbidity and mortality. Pulmonary vein isolation (PVI) is an established procedure for rhythm control in AF. Multiple randomized clinical trials have demonstrated that PVI is a safe therapeutic method and is superior to antiarrhythmic drug therapy in patients with AF, particularly with regard to improvement in symptoms, exercise capacity, and quality of life.^1,2^

The first published data for PVI using cryoballoon (CB) systems was available in 2007.^3^ Considerable effort has been directed towards developing technologies to achieve more effective and durable PVI. While the first-to-market CB (Arctic Front, Medtronic, Minneapolis, MN, USA) is now in its fourth generation,^4^ another novel CB system was introduced in 2020, PolarX, (Boston Scientific, Marlborough, MA, USA).^5–8^

Cryoballoon modalities are thermal-based and have certain limitations in terms of energy transfer and associated complications. While the risk of oesophageal fistula using CB seems to be very low as recently shown in the Potter AF registry (0.0015%),^9^ the incidence of phrenic nerve injury during CB-based PVI was 4.2% in a large multicentre registry of 17 356 patients.^10^ A meta-analysis of 18 studies found sex-specific differences in phrenic nerve injury with an incidence of 3.7% in women and 2.3% in men.^11^ In addition, recurrence of AF after ablation remains common, due to other non-pulmonary vein (PV) triggers^12^ and/or possibly due to failure to achieve durable lesions around the PV ostia.

One of the most significant recent advances to potentially overcome the shortcomings of currently available thermal ablation sources has been the development of pulsed field ablation (PFA). Since PFA appears to exhibit a higher degree of tissue selectivity as suggested by previous studies,^13,14^ in theory it could be safer than CB or radiofrequency, but further studies are necessary to assess its safety in clinical practice. It may also produce more durable lesion and therefore might be more effective. However, whether PFA provides effectiveness similar to a novel CB system in a real-world cohort of patients is unknown. Cryoballoon is the most frequently used single shot technology and hence is the benchmark for PFA.^15–17^

The aim of this study was to compare PFA vs. a novel CB system for PVI regarding efficiency, safety, and follow-up in patients with symptomatic AF.

## Methods

### Patient population

Consecutive patients from the SWISS-AF-PVI registry (NCT03718364) from December 2020 to March 2023 with paroxysmal or persistent AF that underwent first-time PVI using PFA or CB were enrolled. Exclusion criteria included redo procedures or patients undergoing additional lesions such as posterior wall isolation, roof lines, or cavotricuspid isthmus ablation. All patients had to be 18 years or older and provide written informed consent. The choice of ablation system was dependent on logistical reasons. Patient characteristics, anatomical criteria, or patient preference did not affect the choice of modality. The study was carried out according to the principles of the Declaration of Helsinki and was approved by the local ethics committees.

### Procedures

#### Pulmonary vein isolation

The interventions were carried out by four different electrophysiologists, each with prior experience using both modalities. The ablation procedure was performed as previously described^18,19^ with the patient under conscious sedation using midazolam, fentanyl, and propofol. All patients underwent preprocedural imaging of the left atrium via computer tomography or magnetic resonance imaging. Femoral access was performed ultrasound-guided. Transseptal puncture was performed under fluoroscopic guidance. The activated clotting time was kept at a target of 350 s using intravenous heparin. The intracardiac electrograms and surface electrograms were recorded at a speed of 100 mm/s (Sensis, Siemens, Erlangen, Germany). Pulmonary vein isolation was performed using PFA or CB as described below. Technical characteristics of both systems are summarized in Supplementary material online, *Table S1*.

#### Pulsed field ablation

The FARAPULSE Pulsed Field Ablation System (FARAPULSE, Boston Scientific, Natick, MA, USA) consists of (i) a generator (FARASTAR), which produces short, high voltage unipolar pulses; (ii) a 12F over-the-wire multipolar ablation catheter (FARAWAVE) available in sizes of 31 and 35 mm; and (iii) a 13-F inner diameter steerable sheath (FARADRIVE). The design of the ablation catheter features 5 splines containing 4 electrodes each, accounting for a total of 20 ablation electrodes. The five splines can take any form between a spherical ‘basket’ shape to a fully-deployed ‘flower’ configuration. After transseptal puncture an Amplatz Extra-Stiff (0.035”) wire with initially a straight tip and later a J-tip was used to cannulate the veins and the device was deployed inside the left atrium. Pulmonary vein isolation was performed with four PFA applications in basket configuration and four PFA applications flower configuration per vein. Between pairs of PFA applications, the catheter was rotated by 30–40° after two consecutive applications in each configuration, in order to uniformly cover the entire circumference. Ablations were performed using 2 kV.

#### Cryoballoon ablation

The PolarX Cardiac Cryoablation System (Boston Scientific, Natick, MA, USA) consists of (i) a generator (SmartFreeze Console); (ii) a CB (PolarX) available in size of 28 mm with a long tip of 12 mm or short tip of 5 mm (for the purpose of this comparison, only the short tip version was used) and (iii) a 12-F steerable sheath (Polarsheath) with an 12.7 F inner and 15.9 French outer diameter. Maximum deflection of the sheath is 155°. An octapolar inner lumen spiral mapping catheter (PolarMap) was used to visualize PV signals during ablation. After obtaining PV occlusion by optimal alignment of the sheath and the catheter and confirmation by contrast injection, a freezing cycle with a standard duration of 180–240 s was started. Freezing cycles were prematurely terminated in case of phrenic nerve injury. Target temperatures were −40°C and/or PV isolation (time to isolation) within 60 s.^20^ Cryodosing was based on the time to isolation: If PV isolation was within 60 s a 180 s freeze was applied, otherwise 240 s. Applications were prematurely terminated at a temperature of −70°C. No ‘bonus’ freezes were applied after successful isolation of a PV. Entrance block was defined as by elimination of all PV potentials on the octapolar inner lumen spiral mapping catheter. No three-dimensional (3D)-electroanatomical mapping system was used to confirm PVI. Exit block was not routinely assessed in all patients.

### Phrenic nerve injury

Phrenic nerve injury (PNI) was monitored in both groups by pacing the right phrenic nerve (PN) constantly from the right subclavian vein. The PN function was monitored with palpation and with the previously described simple monitoring strategy using the lead augmented vector foot of the 12-lead standard surface electrocardiogram (ECG) for compound motor action potential (CMAP) decrease measurement to monitor diaphragmatic activity and prevent PNI.^21^ In the PFA group, the PN was paced at two time points: prior and after PVI of the right sided PVs. In the CB group, while freezing at the right PVs, the right PN was constantly paced, in case of reduction of PN function (CMAP decrease >30%), the freezing cycle was immediately terminated. PN palsies (PNPs) were defined as PNI persisting at the end of the procedure.

### Procedural endpoint

The procedural endpoint was PVI and was assessed at the end of the procedure for all veins without a waiting period. While in the PFA group a 3D-electroanatomical mapping system (EAM, CARTO3, Biosense Webster, Irvine, CA, USA) was used to confirm PVI at the end of the procedure, in the CB group the spiral mapping catheter (Boston Scientific) was used for endpoint verification. First pass isolation (FPI) was defined in the PFA group, if no residual or recovered PV conduction was noted via EAM. In the CB, FPI was assessed via entrance block confirmation using the spiral catheter in antral position after each application.

### Post-ablation management

Oral anticoagulation was continued for at least 2 months. Hemostasis was reached in both groups via a figure of 8 suture, followed by 6 h of bed rest. Transthoracic echocardiography was performed within 1 h after the end of the procedure to exclude pericardial effusion.

### Endpoints and follow-up

The primary (efficacy) endpoint of the study was freedom from recurrence during 1-year follow-up. After a blanking period of 3 months, any documentation of an AF or atrial tachycardia episode lasting >30 s was counted as recurrence. Arrhythmia in the blanking period that persisted longer than the three blanking months were documented with the earliest occurrence of the arrhythmia. Secondary endpoints (efficiency) were the procedural duration from puncture of the groin to removal of the sheath (procedure duration), duration of left atrial (LA) dwell time and presence of isolation of the PV during first encirclement (FPI). Safety endpoints included phrenic nerve injury, pericardial tamponade, transient ischaemic attack/stroke or vascular access complications. Follow-up including physical examination, 12-lead ECG and 7-day Holter were performed at 3, 6, and 12 months. The same follow-up was applied for patients with cardiovascular implantable electronic devices, except for patients with implantable loop recorders: In these patients remote monitoring was used for arrhythmia detection. In case of symptomatic recurrence outside these planned outpatient clinic visits, a 12-lead ECG or Holter ECG was performed to document the arrhythmia.

### Statistical analysis

Continuous variables are presented as mean and standard deviation (SD) or median (interquartile range), and categorical variables as numbers and percentages. *T*-test was used for continuous, normally distributed variables. Categorical variables were compared using *χ*^2^ or Fisher’s exact test as appropriate. The learning curve was assessed using linear regression modelling with the total intervention duration and left atrial dwell time as the respective outcome variables. For predictors, we considered the number of performed procedures and the energy modality (CB or PFA). For the comparison of recurrences, we used the proportional test. Survival analyses according to Kaplan–Meier were performed and compared using the log-rank test. All analyses were two-tailed, and *P*-values of <0.05 were considered statistically significant. All statistical analyses were performed using R version 4.3.0 (R Foundation 180 for Statistical Computing, Vienna, Austria) with RStudio (version 2023.03.1).

## Results

### Baseline characteristics

Among 223 patients, 42 patients (16%) were excluded because of redo procedures, leaving 181 patients for the final analysis. Of the 181 patients, 106 (59%) patients underwent PVI in the PFA group and 75 (41%) patients underwent PVI in the CB group.

The mean age was 64 (±9.7) years, 64% were male and 64% had paroxysmal AF. The baseline characteristics of the PFA group and the CB group are shown in *Table 1*. The two groups were comparable with no significant differences except for the prevalence of hypercholesterolaemia.

**Table 1:** Baseline characteristics between cryoablation and PFA

Baseline characteristics	CB, *N* = 75	PFA, *N* = 106	Overall, *N* = 181	*P*-value
Age, years	63.5 (9.97)	64.9 (9.42)	64 (9.7)	0.393
Sex (male)	48 (64%)	67 (63%)	115 (64%)	1
BMI, kg/m^2^	27 (24–29)	27 (25–30)	27 (24–30)	0.484
AF type				0.444
paroxysmal	51 (68%)	65 (61%)	116 (64%)	
Persistent	24 (32%)	41 (39%)	65 (36%)	
Left ventricular ejection fraction, SD	58 (9.1)	57 (8.6)	58 (8.8)	0.339
Left atrial diameter, mm, SD	40 (6.3)	41 (6.4)	40 (6.4)	0.221
EHRA score %				0.455
I	10 (13%)	24 (22%)	34 (19%)	
IIa	21 (28%)	33 (31%)	54 (30%)	
IIb	25 (33%)	29 (27%)	54 (30%)	
III	12 (16%)	14 (13%)	26 (14%)	
IV	4 (5.3%)	5 (4.7%)	9 (5%)	
Hypertension	35 (46.7%)	64 (60.4%)	99 (45%)	0.094
Diabetes	6 (8.0%)	12 (11.3%)	18 (9.9%)	0.629
Hypercholesterolaemia	20 (26.7%)	45 (42.5%)	65 (36%)	**0**.**043**
Coronary artery disease	6 (8.0%)	9 (8.5%)	15 (8.3%)	1
Smoking				0.616
Current	9 (12.0%)	11 (10.4%)	20 (11%)	
Never	30 (40.0%)	48 (45.3%)	78 (43%)	
Past	36 (48.0%)	44 (41.5%)	80 (44%)	
Hs-cTnT prior to PVI	8 (6–12)	10 (7–15)	9 (6–13)	**0**.**002**
Available imaging				0.42
CT	7 (9.3%)	15 (14%)	22(12%)	
MR	68 (91%)	89 (84%)	157(87%)	

AF, atrial fibrillation; BMI, body mass index; CB, cryoballoon; EHRA, European Heart Rhythm Association; HS-cTnT, high-sensitivity cardiac troponin T; MR, magnetic resonance imaging; PFA, pulsed field ablation; PVI, pulmonary vein isolation; SD, standard deviation. **Bold**, statistical significance.

### Procedural characteristics

Procedural data are summarized in the *Graphical Abstract* and *Table 2* with 426 PVs and 299 PVs in the PFA and the CB group, respectively. In the PFA group, there were three left common PVs (2.8%) and five right middle PVs (4.7%). In the CB group, there were four left common PVs (5.3%) and three right middle PVs (4%). There was no difference regarding PV anomalies between the groups (*P* = 0.62).

**Table 2:** Comparison between the procedural characteristics

	CB, *N* = 75	PFA, *N* = 106	Overall, *N* = 181	*P*-value
Total procedure duration	58 (48–69)	55 (43–64)	56 (45–66)	0.087
LA dwell time, min	37 (31–48)	38 (30–49)	37 (30–48)	0.871
Fluoroscopy time, min	11 (8.7–16)	11 (9.3–14)	11 (9.2–14.1)	0.814
Hs-cTnT 1 day after PVI	989 (745–1380)	1520 (996–1940)	1160 (856–1730)	**<0**.**001**
Number of applications	5 (4–7)	32 (32–34)		
First pass isolation	42 (56%)	88 (83%)	130 (72%)	**<0**.**001**

CB, cryoballoon; Hs-cTnT, high-sensitivity cardiac troponin T; LA, left atrium; PFA, pulsed field ablation. **Bold**, statistical significance.

Both groups demonstrated a similar procedure duration [55 (43–64) min for PFA and 58 (48–69) min for CB, *P* = 0.087] and left atrial dwell time [38 (30–49) min for PFA and 37 (31–48) min for CB, *P* = 0.871]. Also, the fluoroscopy times were similar with 11 (9.3–14) min for PFA and 11 (8.7–16) min for CB, *P* = 0.814.

Sinus rhythm (SR) was achieved in 100% of cases in both groups and PVI was achieved in 100% in the PFA group and 99.6% in the CB group [due to PNI during ablation of the right inferior PV (RIPV) in one patient]. First-pass isolation was achieved in 88 (83%) in the PFA group and in 42 (56%) in the CB group, (*P* < 0.001). In the PFA group, a median of 32 (32–34) applications were performed per procedure compared with 5 (4–7) freezing cycles in the CB group. In both groups, all patients were in SR after ablation (100%).

**Figure 1:**
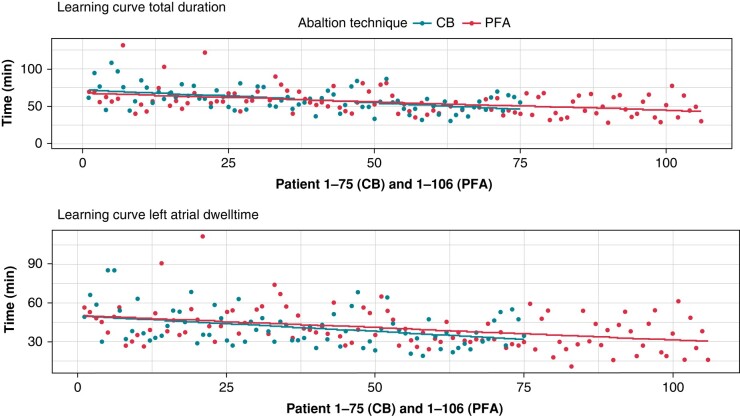
Simple scatter plot representing total procedure duration and left atrial dwell-time over time. The validity of this stratification was confirmed by linear regression modelling. CB, cryoballoon; PFA, pulsed field ablation.

To assess the impact of a potential training effect, the PFA and CB groups were investigated over the time course of the study. Over time, procedure duration and left atrial dwell time decreased in both groups. ‘The significant trend of the learning curve was confirmed using linear regression models: for total procedure duration [intercept of 69 min, −0.25 min/procedure (*P* < 0.001) and change in slope 0.5 for the type of energy (PFA) (*P* = 0.835)] and for left atrial dwell time [intercept of 48 min, −0.20 min/procedure (*P* < 0.001), and change in slope 2.5 for the type of energy (PFA) (*P* = 0.246), *Figure 1*]. First-pass isolation was similar over time with 85% in first 35 and 86% in the following 71 PFA interventions (*P* = 1.00) and 60% in the first 25 and 58% in the following 50 CB interventions (*P* = 0.63).

Myocardial injury, measured by high-sensitive cardiac troponin T (hs-cTnT) the morning after the procedure was significantly higher in the PFA group with a median of 1520 (996–1940) ng/L compared with 989 (745–1380) ng/L in the CB group, *P* < 0.001.

### Procedural safety

Procedural complications are summarized in *Table 3*. Three complications occurred in each group, *P* = 0.7. In the PFA group, two patients suffered cardiac tamponade which required percutaneous drainage and one patient had transient ST elevation after transseptal puncture and before any ablation, probably due to an air embolism. The ST elevations resolved after 20 min and were without further adverse sequelae. All three complications in the CB group were phrenic nerve palsies, two after cryoablation of the right superior pulmonary vein and one after ablation of the RIPV. All phrenic nerve palsies recovered during follow-up, two recovered within 6 months after ablation and one within 12 months after ablation.

**Table 3:** Comparison between the complications

	CB, *N* = 75	PFA, *N* = 106	Overall, *N* = 181	*P*-value
Overall	3	3	6	0.69
PN palsy	3	0	3	0.069
Stroke	0	0	0	
Tamponade	0	2	2	0.512
Vascular access complications	0	0	0	
Other^a^	0	1	1	1

CB, cryoballoon; PFA, pulsed field ablation; PN, phrenic.

^a^Temporary ST elevation.

### Follow-up and atrial fibrillation recurrence

**Figure 2:**
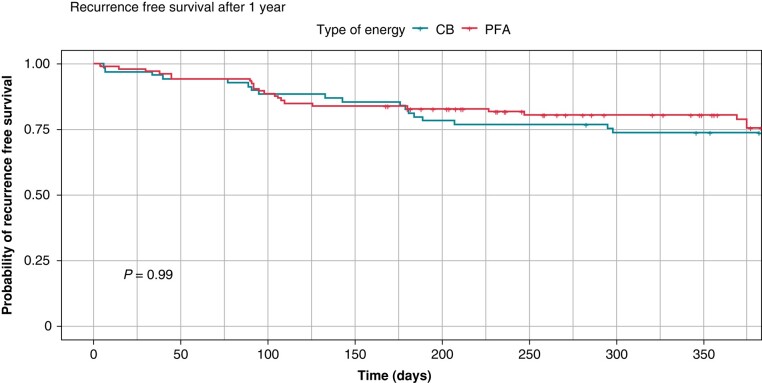
Comparison of Kaplan–Meier curves for recurrence-free survival in the first 365 days after PVI for CB and PFA. CB, cryoballoon; PFA, pulsed field ablation.

Among 181 patients, 6 patients (3.3%) were lost to follow-up. The median follow-up duration was 404 days (208–560). Overall, there were 25 (24%) AF recurrences in the PFA group and 21 (30%) AF recurrences in the CB group, *P* = 0.406. Overall freedom from AF was 81 (76%) for PFA and 48 (70%) for CB (*Figure 2*). In patients with paroxysmal AF, freedom from AF was 48 (74%) and 33 (70%). Twenty patients underwent redo-procedures, which showed 19/23 (83%) durably isolated PVs in the PFA group and 40/55 (73%) durably isolated PVs in the CB group.

## Discussion

In this prospective single-centre study, we aimed to evaluate the procedural characteristics, safety, and 1-year outcome of 181 patients who underwent PVI using either PFA or a novel CB system. We present the following major findings:

First, PFA and CB are very effective for acute isolation of PVs with 100% acute success rate in both groups. Second, AF free survival was ∼75% after 1 year and similar in both groups. Third, procedural efficiency was high and decreased over time with a median procedure time of <1 h in both groups. Fourth, complications were similar in both groups, while phrenic nerve injury only occurred in the CB group. Finally, hs-cTnT levels were significantly higher after PFA than after CB, reflecting larger myocardial damage after PFA.

Our study corroborates and extends previous studies assessing and comparing different modalities for PVI.^16,22,23^ The reported AF free survival aligns with previous studies regarding CB^24,25^ and PFA.^26,27^ Furthermore, to the best of our knowledge, our study is the first to compare PFA with a novel CB system. Prior studies focused on the comparison between RF energy and CB. A meta-analysis of four randomized controlled trials summarizing a total of 1246 patients revealed no difference between RF and CB with regards to freedom from AF at 1 year after ablation (72% for the CB vs. 69% for RF ablation) or with regards to complications.

Recently, Urbanek *et al*.^28^ reported a comparison of CB using the second-generation CB (CB2, 28 mm Arctic Front Advance; Medtronic) with PFA in a single centre retrospective study. They found that PFA compared with CB for PVI showed similar procedural efficacy but was associated with shorter procedure times and no phrenic nerve palsies. Clinical success rates after 1 year were similar compared with our study with no difference between PFA and CB. The limitation of the study by Urbanek *et al*. included the use of an older CB version. Mojica *et al*.^29^ compared the PolarX™ to the Arctic Front™ CB system and found shorter procedure times and fluoroscopy times for the PolarX™ CB system, which could partially also explain the similar procedure time in our study between the PolarX™ CB system and PFA. Further randomized clinical trials comparing PFA with the newest generation CB for PVI are needed and ongoing [Single Shot Champion Trial (NCT0553458: SINGLE SHOT CHAMPION) comparing the FARAPULSE PFA system with the fourth generation CB system (Arctic Front, Medtronic)].

Our study highlights the similarity between PFA and CB regarding procedural characteristics and success rate. Besides these observed similarities, the following differences might impact the choice of modality in the future: first, since a 3D-electroanatomical mapping system was used for the PFA cases in this study, it is expected that the procedure duration and LA dwell time in the PFA group will further decrease when omitting the mapping system. However, whether this has an impact on efficacy needs further investigation. Second, while complications occurred in both groups, PNP only occurred in the CB group. All PNP recovered within 12 months. However, cardiac tamponade can be lethal.^30^ The two cardiac tamponades in the PFA group occurred in 5/106 patients when initially using a stiff guidewire with a straight tip (instead of a J-tip) to cannulate the PV. Thus, from our point of view the complications in the PFA group were not PFA-specific, potentially still promising a safety bonus for the PFA modality. Third, while there is only pilot data available for PFA, there is extensive evidence available regarding the efficiency, safety, and efficacy of CB ablation for AF, including three randomized trials.^1,2,31^ Fourth, vagal denervation seems to occur less often with PFA compared with RF or CB, probably impacting long-term AF free survival.^32^ In this study, there was no difference in AF free survival in patients with paroxysmal or persistent AF. Further studies assessing the mechanism and effect of PFA on the adjacent intrinsic cardiac autonomic nervous system are warranted. Fifth, while the use of CB beyond PVI has been described in case series^33^ and mainly focused on additional isolation of the left atrial appendage,^34^ the versatility of the PFA system, although with only little evidence available, seems to be better suited for posterior wall isolation^35^ or the recently proposed approach of ablation of atrial low voltage myocardium for scar homogenization.^36^

### Limitations

We acknowledge several limitations in this study. First, this was a single-centre, non-randomized comparison of patients participating in a prospective registry. However, although a selection bias may exist, patient characteristics were similar between groups. Second, we did not uniformly use implantable loop recorders in all patients, which is currently considered the gold standard for rhythm monitoring in clinical trials after AF ablation.^37,38^ Third, since PFA is relatively new and currently has only one commercially available catheter, the optimal number of applications and type of lesion set is not known. Further refinement might potentially improve the effectiveness of PFA for PVI. Fourth, information on PV reconnection patterns is limited to patients with recurrence of AF undergoing a repeat procedure. Fifth, very recently, a new CB (PolarX Fit, Boston Scientific) was introduced and received CE marking in April 2023. This novel CB offers a dual diameter balloon size in one catheter to deliver an individualized fit. Further studies comparing PFA with the novel PolarX Fit are warranted.

## Conclusions

Pulsed field ablation and CB were highly effective and efficient for PVI in patient with AF with mean procedure times below 1 h. Procedural characteristics were very similar between both groups in regard to procedure duration, LA dwell time and fluoroscopy duration. While reversible phrenic nerve injury only occurred in the CB group, overall complication rates were similar. Atrial fibrillation free survival after 1 year did not differ.

## Supplementary Material

euad329_Supplementary_Data

## Data Availability

Data are available from the authors upon request.
