# Supplementary material for: Efficacy and safety of pulmonary vein isolation with pulsed field ablation vs. novel cryoballoon ablation system for atrial fibrillation
Source: Europace. 2023 Nov 30;25(12):euad329. doi: 10.1093/europace/euad329 (PMC10751845; doi:10.1093/europace/euad329)
Supplement: euad329_Supplementary_Data [file euad329_supplementary_data.docx]

**Supplementary Data**

|  | **PFA (FARAPULSE)** | **PolarX (Boston Scientific)** |
| --- | --- | --- |
| Ablative energy | Short, high voltage pulses | Cryothermal |
| Energy delivery | 20 electrodes | Whole Balloon surface |
| Deployed size (diameter) | Standard 31mm (flower) | 28mm |
| Electrodes for electrogram recording and pacing | 5 electrodes of 2 mm size | 8 electrodes of 1mm size |
| Inter-electrode spacing | 17 mm (standard) | 6mm |
| Typical duration of a single application | 2.5 sec with 5 pulses | 180-240 sec |
| Typical count application per PV | 4x “Flower” configuration  4x “Basked” configuration | One |
| Delivery sheath | 13-F | 15.9-F |
| CE mark | 02/2021 | 01/2020 |
| FDA Approval | Pending | Pending |

Supplementary Table 1 Technical Comparison of the Ablation Systems, Abbreviations: PFA=Pulsed field ablation, PV=Pulmonary Vein, CE= Conformité Européenne, FDA=Food and Drug Administration
